# Medical Library Association Diversity and Inclusion Task Force Report

**DOI:** 10.5195/jmla.2021.1112

**Published:** 2021-01-01

**Authors:** Jane Morgan-Daniel, Xan Y. Goodman, Sandra G. Franklin, Kelsa Bartley, Matthew Nicholas Noe, JJ Pionke

**Affiliations:** 1 morgandanie.jane@ufl.edu, Community Engagement and Health Literacy Liaison Librarian, Libraries, University of Florida Health Science Center, Gainesville, FL; 2 xan.goodman@unlv.edu, Liaison Librarian, Lied Library, University of Nevada, Las Vegas, Las Vegas, NV; 3 librsf@emory.edu, Director, Woodruff Health Sciences Center Library, Emory University, Atlanta, GA; 4 k.bartley@med.miami.edu, Education and Outreach Librarian, Learning, Research & Clinical Information Services Department, Louis Calder Memorial Library, University of Miami Miller School of Medicine, Miami, FL; 5 matthew_noe@hms.harvard.edu, Primary Selector, Health Sciences, and Curator, Specialized Collections, Countway Library, Harvard Medical School, Boston, MA; 6 pionke@illinois.edu, Liaison Librarian, University Library, University of Illinois–Urbana-Champaign

## Abstract

The Medical Library Association (MLA) appointed a Diversity and Inclusion Task Force (DITF) in 2017. Sandra G. Franklin, AHIP, FMLA, chaired the task force and guided initiatives. From 2017 to 2020, the task force completed a review of MLA defining documents—including the mission, vision, values, and code of ethics—resulting in language updates to these documents. As MLA transitioned through the communities process, the DITF contributed to the transition. Other recommended essential changes to MLA profiles to promote awareness included updating pronouns to promote gender inclusivity and suggestions for the Annual Meeting Innovation Task Force. DITF members actively brought diversity and inclusion programming and engagement to MLA members at annual meetings. The task force held a fish bowl conversation, an open forum, and a Diversity Dialogues roundtable discussion; provided interactive discussion boards; and designed an MLA diversity button. Beyond MLA annual meetings, the task force hosted two critical librarianship meetings and a Twitter chat to engage MLA members with diversity and inclusion topics. Task force members promoted diversity and inclusion beyond their task force appointments with presentations at chapter meetings and other non-DITF MLA annual meeting programming. A notable task force accomplishment included completing a survey of MLA members to gather baseline demographic characteristics, including never before collected data about disability, socioeconomics, and caregiver status. This report provides an overview of DITF activities from 2017 to 2020.

## INTRODUCTION

Teresa L. Knott, AHIP, 2016/17 Medical Library Association (MLA) president, announced the Board of Director's decision to make diversity and inclusion a strategic goal of the association in December 2016 [1]. The board identified three essential actions: “draft and consider an MLA strategic goal on diversity and inclusion for roll-out after the MLA '17 ‘Dream Dare Do' meeting in Seattle; organize an open forum on diversity and inclusion during MLA '17; engage our members already active in diversity and inclusion at MLA, and others who are interested in supporting MLA's efforts.” As a result, by May 2017, the board had identified diversity and inclusion as a strategic goal for the organization (supplemental Appendix A: MLA strategic plan with diversity and inclusion goal).

In summer 2017, 2017/18 President Barbara A. Epstein, AHIP, FMLA, issued a call for a Diversity and Inclusion Task Force (DITF). The call for applicants was shared with existing MLA entities such as committees, sections, and special interest groups (SIGs). The open invitation to join the newly created DITF required applicants to describe in 100 words or less 3 of the most important reasons they wanted to participate in the task force. If selected to serve, members were expected to attend the 2018 and 2019 MLA annual meetings [2]. Supplemental Appendixes B and C provide the email call for applications and blog post announcing the task force members from President Epstein.

There were nearly forty applicants, and thirteen were selected to serve on the DITF. By August 2017, the task force was fully appointed with Sandra G. Franklin, AHIP, FMLA, as chair. Tomi Gunn was assigned as the MLA staff liaison, and 2018/19 President Beverly Murphy, AHIP, FMLA, was the MLA Board liaison. Once Murphy began her MLA presidential year, Gupreet Kaur Rana was appointed as MLA Board liaison to the DITF [3]. Over three years (September 2017 to May 2020), the DITF coordinated with MLA membership and leadership to accomplish a number of actions and initiatives. This report summarizes the DITF's activities during this time period. On June 1, 2020, the DITF transitioned into the new MLA Diversity, Equity, and Inclusion (DEI) Committee, chaired by Xan Y. Goodman, AHIP.

## DIVERSITY AND INCLUSION TASK FORCE (DITF) MEMBERSHIP

**Figure 1:**
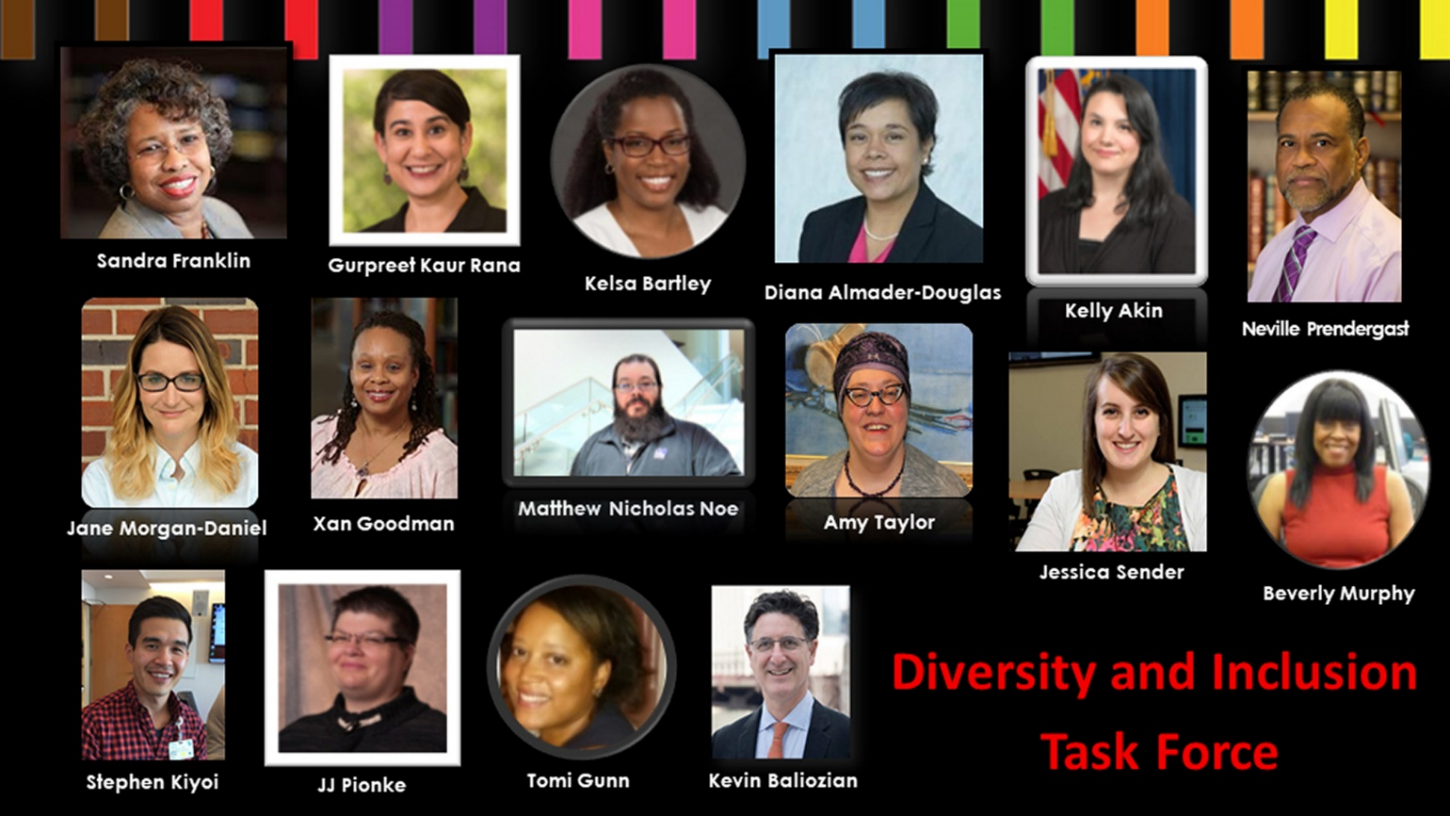
Diversity and Inclusion Task Force (DITF) members shown in a PowerPoint slide by Beverly Murphy, AHIP, FMLA

Chaired by Franklin and comprising twelve members (Figure 1), the DITF formed in 2017 with the purpose of executing MLA's new Diversity and Inclusion strategic goal. To initiate open dialogue before the DITF's inaugural meeting, each member was asked to address the discussion prompt “My idea for diversity and inclusion for MLA is…” (Table 1).

**Table 1:** DITF members' responses to the discussion prompt “My idea for diversity and inclusion for MLA is…”

My idea for diversity and inclusion for MLA is…
“promoting interests & professional development of all underrepresented groups in a manner that is proactive & not reactive”
“all members will know it to be a core value because it is expressed throughout association documentation and expected in the association's leadership”
“recognizing that we must make opportunities for discourse available to [effect] change”
“ensuring underrepresented groups have a voice in our profession”
“fostering awareness, understanding, cultural sensitivity, respect, & responsiveness towards the values & needs of different communities”
“focusing on the whole experience of librarianship, from library assistants to graduate school to professional life…it has to include financial & cultural action”
“where we are aware that diversity is an acknowledgement of our differences & that inclusion means that we embrace them”
“creating an infrastructure to enhance the participation of diverse librarians”

## DITF CHARGE AND GOALS

The DITF's first formal meeting took place in November 2017, during which the charge and goals were reviewed. The charge was to “evaluate and improve MLA practices as they relate to diversity and inclusion” in the following areas:

- MLA defining documents, including vision, mission, values, and code of ethics statements;
- MLA publications, including the *Journal of the Medical Library Association (JMLA)* and *MLA News;*
- MLA programs, including the annual meeting, education, and credentialing;
- formulation of position statements, including scope of issues and processes;
- engagement of members in a constructive discourse on sensitive issues; and
- encouragement of a diverse audience to participate in MLA leadership.

Accordingly, the DITF's five goals were to:

1. build activities and programs that create and sustain diverse, inclusive, and welcoming cultures and practices;
2. ensure that members, volunteers, and staff have a high level of awareness of issues related to diversity and inclusion;
3. ensure that what we do as an organization, and how we do it, reflects the essential values of diversity and inclusion;
4. attract a diverse community of members that reflects the diversity of the profession and those we serve; and
5. apply the best practices of professional associations with regard to diversity and inclusion.

## DITF ACTIVITIES SEPTEMBER 2017 TO MAY 2020

### Review of MLA's vision, mission, values, and code of ethics

The DITF chair identified areas for review and asked for a volunteer subgroup to begin work in May 2018. The subgroup—led by Goodman and composed of Diana Almader-Douglas, AHIP, Jane Morgan-Daniel, AHIP, and JJ Pionke—reviewed MLA defining documents for language inclusivity, including the vision, mission, values, and code of ethics. A review of the grants and scholarships language also took place.

First, the subgroup identified other library-related organizations with potentially relevant diversity documents including the American Library Association (ALA), Association of Academic Health Sciences Libraries (AAHSL), Chartered Institute of Library and Information Professionals (CILIP), European Association for Health Information and Libraries (EAHIL), International Federation of Library Associations and Institutions (IFLA), and Special Libraries Association (SLA).

After reviewing the language used in these organizations' mission statements, the subgroup compiled a report for the DITF chair with recommendations to update and change language in the aforementioned MLA documents. This report resulted in two motions submitted to the MLA Board of Directors in August 2019 that included supporting documents with the sources consulted. Franklin, DITF chair; Rana, MLA Board liaison, and Kevin Baliozian, MLA executive director, all provided input and guidance for the proper structure of the motions. The first motion suggested changes to MLA's vision, mission, code of ethics, and values. The second motion recommended changes to language throughout all MLA documents, for example, changing Hispanic to Latinx. The motions were accepted and passed by the MLA Board on September 3, 2019 (supplemental Appendix D).

The newly established MLA DEI Committee will address further issues related to revising MLA documents, because there was some sentiment that the recommended language did not go far enough to advocate for equity and inclusion.

### MLA profile information changes

Due to the importance of gender identity inclusivity and the use of gender-diverse pronouns, the DITF asked that pronouns be added to the annual meeting badges, as well as the option to choose a gender neutral honorific such as Mx during registration be added (supplemental Appendix E). For 2018, the first year of this change, meeting badges had ribbons to denote pronouns. Starting in 2019, pronouns were added directly to the badges themselves.

### Contribution to communities transition

#### Modeling community changes.

The Communities Task Force spent two years looking at models to evolve MLA community participation. Prior task forces and the Rising Stars had produced recommendations to make the professional home of members more inclusive. Following a presentation by Executive Director Baliozian about the proposed structure of caucuses and domain hubs, the DITF was asked to weigh in. Nine members made comments that were shared with Communities Task Force Chair Rikke Sarah Ogawa, AHIP, in August 2018.

#### Transforming MLA communities.

Detailed information was published in November 2018 with a statement that the two task forces had worked together to agree on the following recommendations approved by the MLA Board:

- replacing the dual-level of MLA member communities (sections and SIGs) with a single tier (caucuses); and
- eliminating the financial barrier to joining an MLA member community by setting all MLA member community dues to $0 [4].

The association implemented these and other changes starting September 1, 2019, with an immediate positive effect on MLA's diversity and inclusion.

### Recommendations to the Annual Meeting Innovation Task Force

The DITF made multiple recommendations for MLA annual meetings, based both on conversations about DEI and on the results of the membership survey that ran in October 2019. Largely, the concerns around the annual meeting centered on cliquishness, cost, communication, and ability to connect with other people during the meeting. The DITF recommendations included creating alternative social activities that did not involve noisy and crowded venues and instead offer alternative activities, like cards, board games, and coloring events; providing more robust support for an array of needs such as gender inclusive bathrooms, a lactation room, a meditation space, a list of food establishments for those with religious observances; and providing other types of support for attendees to make them feel welcome and included.

### Diversity and inclusion guiding principle

The MLA Communities Task Force requested a guiding principle from the DITF to contribute toward affirming DEI as a core organizational value during and after the communities transition process. The statement was created by Rana:

“Diversity, equity, and inclusion are the threads that strengthen the fabric of the Medical Library Association”

### DITF activities at MLA annual meetings

#### MLA '18 open forum.

The MLA '18 “Diversity and Inclusion Strategic Goal Open Forum” was designed to serve the following purposes: enabling the DITF to share their goals and activities with as many people as possible in-person, letting the membership become familiar with DITF members face-to-face, and serving as a follow-up to the interactive discussion boards set up throughout the meeting on which people were asked to share their thoughts on topics such as “what diversity and inclusion means to you” and “how does length of service at MLA relate to diversity and inclusion?” The responses on these boards, up until the time of the open forum, were used as springboards for further discussion.

The open forum was well attended (standing room only), with membership sharing a number of questions and concerns that helped to inform the next steps of the DITF, including calls for benchmarking data, language changes for the MLA Scholarship for Minority Students, and calls for continuing education (CE). Membership rightly discussed how the lack of diversity in librarianship is a larger problem, but as one task force member pointed out, the DITF's scope was to “clean our house first” rather than to try and address diversity and inclusion in the entire field of library and information science (LIS).

In an uncomfortable moment, a member stood up and said that they did not really understand what all of this was about (specifically not understanding the concept of white privilege), and a productive discussion followed. This discussion highlighted several important issues, including that despite master's degree credentialing (among others), the LIS field had not done a sufficient job of educating on DEI matters. The open forum demonstrated that equity and inclusion was an issue that MLA should dedicate time to addressing at an organizational level, above and beyond what was accomplished by the task force. Member comments at the open forum also highlighted an unmet need for more opportunities in MLA for such uncomfortable but essential conversations to happen. While encouraging the creation of resource lists and continual reading and self-education, it is also important to recognize that doing the reading is absolutely necessary but not sufficient on its own. Sometimes the hard conversations must happen. To move toward these hard conversations, the DITF sponsored a diversity fish bowl session at MLA '18.

#### MLA '18 fish bowl.

At MLA '18, a special session was conducted called the “Diversity and Inclusion Fish Bowl.” Thirty-four meeting attendees participated in the one-and-a-half-hour hour session, which was facilitated by Blair Anton, AHIP, Alexa Mayo, AHIP, Anne K. Seymour, and M.J. Tooey, AHIP, FMLA. The objective was to gather participants' thoughts through participatory dialogue. Morgan-Daniel and Amy Taylor recorded responses to three questions:

1. In an ideal world, what would diversity and inclusion look like for MLA?
2. What strategies can MLA develop and adopt to ensure diversity and inclusion in offices, committees, and other leadership positions in the association and its units?
3. What can MLA do to attract and retain members of diverse backgrounds and experiences, and what are strategies for mentoring and encouraging effectively?

Five main themes emerged:

1. **Recruitment and retention:** increasing support for new members, advocating for health sciences curriculums in library schools, conducting outreach to minority groups to encourage them to pursue librarianship as a career, and recognizing the value of non–master's of library science (MLS) skill sets
2. **Organizational structure:** ensuring a diverse MLA Board and leadership, encouraging more SIGs for underrepresented groups, encouraging minority groups to apply for committees and leadership positions, and ensuring that length of time is not the only consideration for appointments
3. **Resources:** recognizing invisible disabilities, supporting innovative programming, conducting ally training, enabling more remote participation, reducing costs of annual meetings, having all-gender restrooms and religious accommodations at meetings, and conducting safe space training
4. **Mentorship:** improving the visibility of MLA's mentoring program, encouraging more minority leaders to mentor, creating a mentoring competency checklist, offering mentorship training, and providing mid-career mentorship
5. **Communication:** limiting use of acronyms to minimize confusion for new members, creating a framework of cultural humility, acknowledging that colorblindness is invalidating, finding ways to encourage dialogue regarding the different backgrounds of members, tailoring meeting event options for both extroverts and introverts, and keeping current with evolving DEI terminology and conversations

#### MLA '19 roundtable.

At MLA '19, the DITF hosted a ninety-minute Diversity Dialogues roundtable. Approximately eighty attendees joined roundtables facilitated by Kelsa Bartley, Franklin, Goodman, Mary Catherine Lockmiller, AHIP, Morgan-Daniel, Pionke, and Rana. Conversation topics included the perceived inclusivity of MLA as an organization in relation to disabilities; lesbian, gay, bisexual, pansexual, transgender, genderqueer, queer, intersexed, agender, asexual, and ally community (LGBTQIA+); and socioeconomic status (SES). Participants were encouraged to share their stories, experiences, and viewpoints in response to the questions (Table 2).

**Table 2:** Diversity Dialogues roundtable questions

Disabilities	LGBTQIA+	Socioeconomic status (SES)
What are the biggest challenges for people with disabilities and how can MLA help?	What are the biggest challenges for LGBTQIA+ people and how can MLA help?	What are some of the socioeconomic challenges MLA members experience?
In relation to people with disabilities and inclusivity, which issues or actions do you feel should be prioritized by MLA and why?	In relation LGBTQIA+ people and inclusivity, which issues or actions do you feel should be prioritized by MLA and why?	What do you think are the biggest challenges related to SES or of being a first-generation librarian in MLA?
Can you recommend disability or accessibility inclusive resources that are useful for learning and teaching?	Can you recommend LGBTQIA+ inclusive resources that are useful for learning and teaching?	Can you recommend resources to learn more about SES and health sciences or medical librarianship?
What are some misconceptions about disability folx that you have encountered as a member of MLA?	What are some misconceptions about LGBTQIA+ folx that you have encountered as a member of MLA?	Can you describe intersections between education, income, and occupation that might affect MLA members?
What positive or negative experiences have you had as a person with disabilities and as an MLA member?	What positive or negative experiences have you had as an LGBTQIA+ MLA member?	What positive or negative experiences have you had as an MLA member related to SES or as a first-generation librarian?
How could MLA leadership enhance the environment for members with disabilities?	How could MLA leadership enhance the environment for LGBTQIA+ members?	Can you describe any dynamics of power in MLA that you have seen or experienced?
What is the question you are most tired of hearing? What would you like to say about it so you never have to answer it again?	What is the question you are most tired of hearing? What would you like to say about it so you never have to answer it again?	Can you describe the relationship between cultural and political capital in MLA? How does this affect members?

The roundtables fostered discussions on negative experiences, misconceptions, and microaggressions. Additionally, a number of suggestions were provided by participants:

1. **Recommendations for annual meetings:** increasing diverse representation on planning committees, developing an inclusive meeting checklist, providing quieter social events and relaxation spaces, ensuring wheelchair accessible seats at the front of meeting rooms, making seating available during beverage breaks, providing a disability services ambassador or accessibility booth, determining registration rates based on salary, increasing meeting scholarships, offering child care facilities, and creating guidelines for presentations on accessibility and inclusive language
2. **Future continuing education (CE) courses and meeting programming topics:** covering invisible disabilities, LGBTQIA+ ally training, implicit bias, vocational awe, microaggressions, inclusive language, privilege in the workplace, and career progression trainings
3. **MLA organizational structure and leadership:** creating a disability caucus, checking whether the MLA website complies with Web Content Accessibility Guidelines, creating a DEI statement, ensuring diverse representation of leadership, conducting LGBTQIA+ Safe Zone Project training for all leaders, and providing more remote engagement opportunities because many members cannot afford to attend meetings

### #MLADiversity hashtag

**Figure 2:**
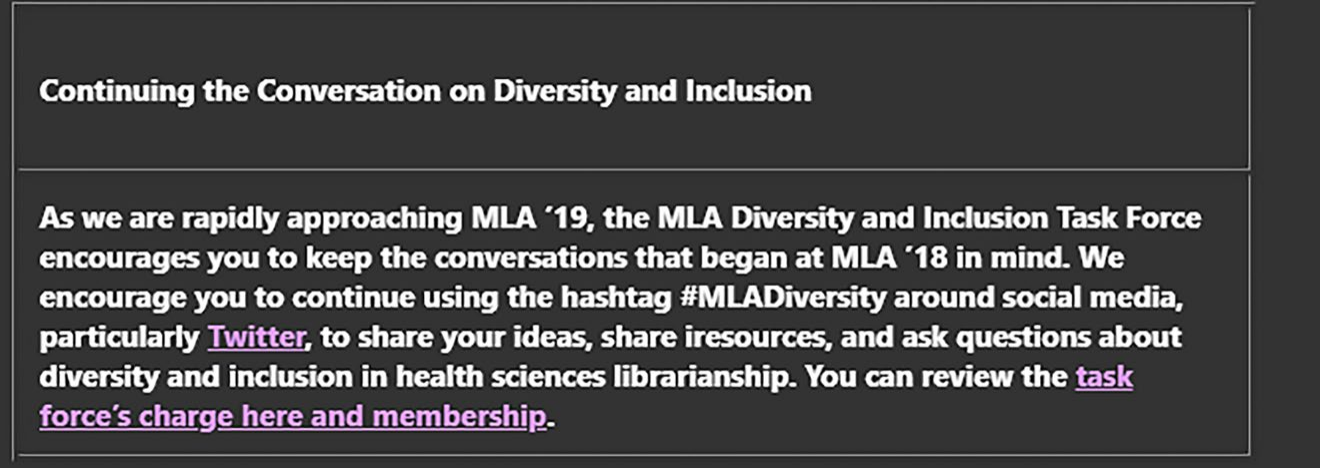
DITF blog post publicizing the Twitter hashtag

**Figure 3:**
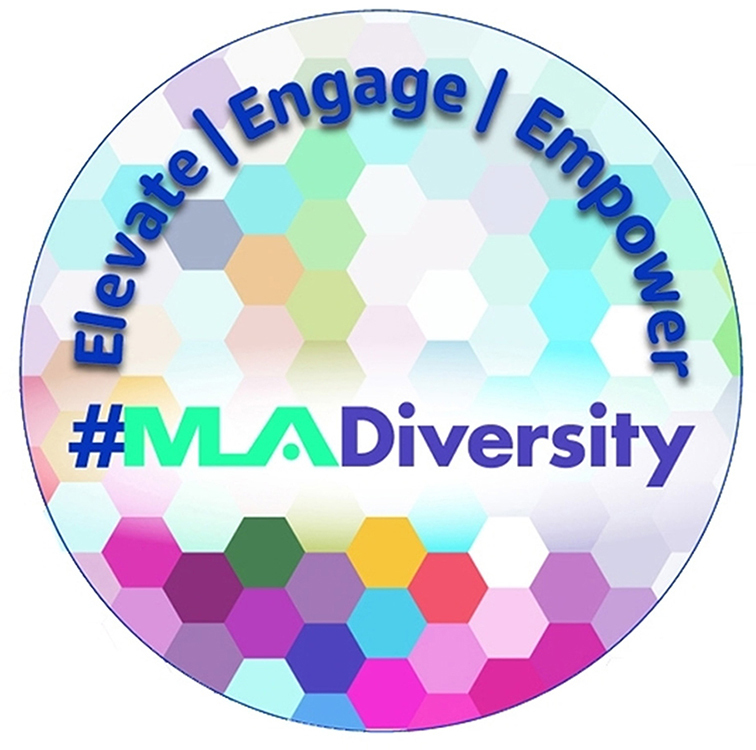
MLA '18 diversity button design

An #MLADiversity Twitter hashtag was conceived and adopted by the DITF in spring 2018. It was developed to initiate and capture conversations on Twitter around issues and topics related to DEI in medical librarianship and beyond. The hashtag was first used during MLA '18 to broaden the discussion during the open forum. It was publicized beyond Twitter in blog posts by DITF members for MLANET and highlighted in *MLAConnect* (Figure 2). Two DITF members, Bartley and Matthew Nicholas Noe, were charged with coming up with suggestions for the hashtag and were official tweeters for the meeting that year, assisting with spreading the use of the hashtag among medical librarians (#medlibs) on Twitter. DITF members wore buttons at MLA '18 with the hashtag (Figure 3). The button was designed by Murphy, Gunn, and other MLA staff.

The hashtag has been used more than 500 times between May 2018 and June 2020, as the promotion and use of #MLADiversity continued beyond MLA '18. For example, the hashtag was used in a #medlibs Twitter chat “Making a Better MLA” on February 14, 2019, which discussed steps and actions to create a positive and welcoming environment for all, as well as strategies for countering negative situations in the profession. In March 2020, the DITF held its first Twitter chat, which was organized and hosted by the DITF Communications Subgroup members Goodman and Bartley, and facilitated on Twitter by Gunn and Martha Lara from MLA staff. The chat focused on institutional activities and personal development relating to DEI, so that members could engage and share their personal journeys, ideas, and information on what their libraries and organizations were doing to further DEI efforts. Four questions were discussed during the hour-long chat:

1. What is your institution actively doing to promote DEI?
2. Are you involved in DEI activities at your institution or library? What kinds of activities are you involved in?
3. What are you personally doing to improve your understanding and awareness about DEI? (What are you reading, watching, intending to learn)?
4. Engaging with DEI topics or discussing DEI can be challenging. How do you find support if you experience discomfort or stress related to these topics?

Approximately twenty to thirty people participated from across the country. Selected mentions are listed below.

**On institutional activities:**
- DEI book clubs, discussions, a diversity hiring recommendations report
- New, higher level division devoted to diversity and inclusion, every unit writing diversity statements, a new tribal liaison librarian
- Educational series in a cohort style, group meetings of faculty and staff to discuss DEI
- Formation of DEI task forces or committees
- Hiring a DEI librarian, sponsoring activities and education, collaborations on campus, commitment to recruiting and retaining diverse candidates, fostering DEI project opportunities
- A chancellor's diversity advisory committee, LGBTQIA+ employee alliance, groups to support minority faculty and students, an appointed director of inclusion

**On personal development:**
- “Trainings from different organizations on topics such as diversity audits of collections.”
- “It simply isn't possible to address all of it everywhere. The problems are too enormous, too complex, too integrated into our own context and identity. We start where we are, and move from there, knowing we are flawed, & so is the process…”
- “Knowing I don't know everything and that is ok…having a growth mindset is important. Realizing that experiences will change your views on things.”

**On discomfort engaging with DEI topics or discussing DEI and finding support:**
- “Depends on the source of discomfort. If it's because of learning new things: sit with it, listen more than you talk, keep learning. If it's because you're part of a marginalized population and being asked to speak for everyone, teach everyone, etc., [defuse] issues, protect yourself.”
- “Welcome the discomfort and stress…it means that's exactly where I need to be so I can grow. I lean into it, but I also like when my mind is blown by new concepts & things I used to believe are found to be false (like my biases).”
- “If you think you're feeling discomfort at educating yourself about #DEI, consider how difficult life is for the marginalized populations and their struggles.”
- “Too often ‘resilience' or ‘grit' is code for ‘we know the system is broken and biased but it's your job to just power through it as-is.'”
- “Finding a sense of community outside the library for these conversations, for example, institutional support such as cultural resource centers, student services professionals, and wellness offices.”

**On suggestions for DEI content at MLA meetings:**
- Training on intervention strategies with various levels of power involved, for example when micro (or macro) aggressions are made, how to stop them and educate someone
- How to engage library personnel who are not supportive of DEI efforts
- More content on disability and accessibility
- An official certificate (like the Consumer Health Information Specialization [CHIS] but for health care) that keeps track of continual learning regarding DEI
- LGBTQIA+ allyship through action, language, or recommended readings
- Recruiting and retaining librarian people of color (POC)

As is often the case with social media, the hashtag has now grown into its own and is used to discuss DEI issues of relevance to medical librarianship well beyond just the “official” use by the DITF and MLA staff. A history of the hashtag can be viewed at https://twitter.com/hashtag/MLADiversity?src=hashtag_click.

### Timeline of DITF activities during MLA '17 to MLA '19

In addition to a proposed motion to create a new DEI Committee within the MLA structure and updates to language in MLA documents, the DITF met their objective to “build activities and programs that create and sustain diverse, inclusive, and welcoming practices” through organized activities that involve MLA membership (Table 3).

**Table 3:** Summary of task force activities

Activity	MLA meeting year
Developed diversity buttons	2017–2018
Investigated MLA defining documents	2017–2019
Held a fish bowl	2018
Held an open forum	2018
Provided interactive discussion boards	2018
Held critical librarianship webinar series	2019
Held diversity and inclusion roundtables	2019
Hosted Twitter chats	2020
Submitted a motion to change language in MLA defining documents	2019
Surveyed MLA membership	2019

DITF members were ardent advocates of DEI and embodied this in their unofficial activities beyond task force membership. A few examples are notable. Bartley led and organized book clubs for MLA members that kept the conversation about diversity and inclusion at the forefront from 2018 to 2020. Pionke took the initiative and created a low stress activity during MLA '19. He brought crayons and sheets of paper and sat in a table area outside of the exhibits hall. Folx joined Pionke to restfully color and engage in conversation. Almader-Douglas designed and presented a poster at the 2018 annual regional meeting of the Medical Library Group of Southern California and Arizona (MLGSCA). Almader-Douglas's poster provided an overview of the formation of the DITF and how efforts of diversity and inclusion fit in the MLA strategic plan, along with the goals and objectives of the task force. Almander-Douglas also provided an overview of task force activities up to that point. Beyond these milestones, Almader-Douglas shared the #MLADiversity hashtag developed by task force members and encouraged MLGSCA members to provide feedback about diversity and inclusion. Finally, Almander-Douglas shared potential next steps that the task force might pursue. These few examples demonstrate the commitment that task force members had toward the topic of DEI.

Other related MLA '18 and MLA '19 programming

Tables 4 and 5 provide summaries of DEI-related presentations at MLA '18 and MLA '19.

**Table 4:** MLA '18 presentations

Title	Authors	Format	Date
Adaptations in Global Health Education: Reinventing a Course Collaboration through a Global Data Lens	Gurpreet K. Rana and Laura S. Rozek	Paper	May 20, 2018
Building an Assistive Technology Workspace	JJ Pionke	Poster	May 22, 2018
Creating a Consortium Task Force to Assess E-Resource Accessibility	JJ Pionke, Elizabeth Sosnowska, and Heidi M. Schroeder	Poster	May 20, 2018
An International Collaboration: Team-Based Informationist and Physician Instruction in Ghana	Emily C. Ginier and Gurpreet K. Rana	Poster	May 20, 2018
Presidential Inaugural Address	Beverly Murphy, AHIP	Speech	May 22, 2018
A Transformative Ghana-US Collaboration: Developing an Innovative Health Sciences Librarian Exchange	Emily C. Ginier and Gurpreet K. Rana	Poster	May 21, 2018
The Transforming Landscape of Cultural Diversity in the Biomedical Literature	Karen Gutzman, Diana Almader-Douglas, Brenda M. Linares, AHIP, Annabelle Nunez, and Bredny Rodriguez, AHIP	Poster	May 20, 2018
Veteran Voices: Library Impact on Veterans	JJ Pionke	Poster	May 22, 2018

**Table 5:** MLA '19 presentations

Title	Authors	Format	Date
Building Capacity in Nigeria to Promote Implementation Health Sciences: A Workshop Experience	Xan Y. Goodman, AHIP	Poster	May 7, 2019
Clueless at MLA: New Member Immersion Session	Nisha Mody, Keith Engwall, AHIP, Erin E. Reardon, Kelsa Bartley, Jahala Simuel, Alexandria Leigh Brackett, AHIP, Alice Jean Jaggers, Siobhan Champ-Blackwell, Jolene M. Miller, Nisha Mody, and Stacey Arnesen	Immersion session	May 5, 2019
Connecting Consumer Health Information with Clinical Care	Hannah F. Norton, AHIP, Margaret Emily Ansell, AHIP, Matthew Daley, Mary Edwards, and Jane Morgan-Daniel, AHIP	Paper	May 5, 2019
Disability and Accessibility: Training Needs of Librarians	JJ Pionke	Poster	May 6, 2019
Disability and Accessibility: Understanding the Education Needs of Library Graduate Students	JJ Pionke	Poster	May 7, 2019
On the Same Page: Aligning Librarians' and Requestors' Relevance Perceptions to Improve the Search-and-Weed Process	Jane Morgan-Daniel, AHIP, Nancy Schaefer, AHIP, Linda Struckmeyer, Luther King, Christine T. Myers, Shabnam Medhizadah, Mary Jeghers, and Jason Beneciuk	Lightning talk	May 6, 2019
Raising Awareness of Diversity: One Chapter's Experience	Brenda M. Linares, AHIP, Beverly Murphy, AHIP, FMLA, Tony Nguyen, AHIP, Ene O. Belleh, AHIP, and Carenado Davis	Paper	May 7, 2019

### Continuing education (CE)

CE related to diversity themes was offered at MLA annual meetings in 2018 and 2019. These efforts focused on implicit bias training and microaggressions (Table 6).

**Table 6:** Continuing education

Title	Authors	Presentation format	Dates
Implicit Bias Training for Information Professionals	Shannon D. Jones, AHIP, Kelsa Bartley, and Kimberly L. Reynolds	Continuing education course	May 4, 2019
Microaggressions and More: Continuing the Conversation on Implicit Bias	Andy Hickner, Diana Almader-Douglas, AHIP, James Eddy Anderson, Shannon D. Jones, AHIP, Brenda M. Linares, AHIP, Hannah Rutledge, AHIP, Megan Threats, and Tara Douglas-Williams, AHIP	Immersion session	May 7, 2019
Transforming Libraries Using Implicit Bias Training	Kimberly L. Reynolds, Shannon D. Jones, AHIP, and Kelsa Bartley	Special content session	May 20, 2018

#### MLA '18 implicit bias immersion session.

At MLA '18, the African American Medical Librarians Alliance (AAMLA) SIG sponsored the special content session, “Transforming Libraries Using Implicit Bias Training,” presented by Kimberly L. Reynolds, assistant professor of clinical pediatrics, University of Miami Miller School of Medicine and Pediatric Hospitalist, Holtz Children's Hospital at Jackson Memorial Medical Center. The event was organized and moderated by Jones and Bartley.

The ninety-minute interactive session provided an overview of implicit bias, the impact it has in libraries and in health care, and how it stands in the way of diversity and inclusion. An implicit bias is when individuals have attitudes toward people or associate stereotypes with them without their conscious knowledge. Accordingly, the main goals of the session were to help attendees:

- increase their awareness of implicit bias and the role it plays in the work setting
- learn how implicit bias can hinder or enhance the overall effectiveness of libraries
- gain an understanding of how implicit biases impact hiring decisions
- gain practical strategies to help attendees uncover and work through their own biases

Continued interest in the topic and feedback received after this initial session led to the development of future programs and sessions, including:

- “Microaggressions and More: Continuing the Conversation on Implicit Bias” immersion session at MLA '19
- “Implicit Bias Training for Information Professionals” CE course at MLA '19, which was also presented at the Upstate New York and Ontario Chapter (UNYOC) Annual Chapter Meeting in October 2019 and at the University of Miami Libraries in March 2020
- MLA Reads Virtual Book Discussion Group 2018–2020

#### Serving Patrons with Disabilities in Your Library or Clinic webinar.

A continuing education webinar was proposed by Christine Willis, AHIP, on the topic of accessibility and disability in libraries, “Serving Patrons with Disabilities in Your Library or Clinic,” and was held April 17, 2020.

#### “Microaggressions and More: Continuing the Conversation on Implicit Bias” immersion session.

The “Microaggressions and More: Continuing the Conversation on Implicit Bias” immersion session was held at MLA '19. It was organized by Andy Hickner and the Social Justice Section in collaboration with several MLA communities and members:

- MLA sections and SIGs involved: AAMLA SIG, Health Disparities, Latinx SIG, Leadership and Management Section, LGBTQIA+ Health Sciences Librarians SIG, and Social Justice Section
- MLA members and DITF members involved in organizing this event: Almader-Douglas, James Eddy Anderson, Bartley, Patricia Devine, Tara Douglas-Williams, AHIP, Hickner, Shannon D. Jones, AHIP, Brenda M. Linares, AHIP, Robert T. Mackes, AHIP, Hannah Rutledge, AHIP, Gregg A. Stevens, AHIP, Susan E. Swogger, and Megan Threats
- The panel discussion was moderated by Linares, and the panelists were: Jones, Almader-Douglas, Rutledge, Anderson, and Threats

As part of an ongoing series of MLA programs exploring the topic of implicit bias, the session continued the conversation started with the MLA '18 special content session, “Transforming Libraries Using Implicit Bias Training.” The highly interactive session focused on the concept of microaggressions, defining the term, providing concrete examples of microaggressions in day-to-day professional lives, recognizing when individuals themselves may intentionally or unintentionally exhibit these behaviors, and sharing techniques for responding to them. Microaggressions researcher Threats discussed implicit bias in relation to providing health information services to diverse populations, sharing evidence on how microaggressions and implicit biases impact patients with marginalized identities. The panelists shared their personal and professional experiences with implicit bias and microaggressions. Audience participation during the session included questions for the panelists and the opportunity for audience members to share their own perspectives.

#### Critical librarianship webinars.

At MLA '19, DITF members discussed offering a series of free webinars to introduce critical librarianship to health sciences librarians, as Jill Barr-Walker and Claire Sharifi had recently published a *JMLA* article about critical librarianship that provided a good overview [5]. Task force members brainstormed how to best introduce this topic and decided to invite Barr-Walker and Sharifi to present the first webinar, along with other librarians who would discuss their journeys with critical librarianship. The additional invited librarians were Nisha Mody and Bethany Myers, AHIP. The first webinar was held August 14, 2018. The second webinar featured Pionke, Noe, and Jones on October 21, 2019. Pionke discussed his work related to disabilities in LIS and shared personal stories from his institution. Noe discussed how graphic medicine can be a departure point for using library collections to encourage social justice conversations. Noe also shared his personal experience with social class in librarianship. Jones shared how she used critical librarianship practices as a director to cultivate and lead a library that was welcoming, safe, and inclusive. Both of these webinars were widely attended by MLA members, with over 140 people at each session. As a result of these webinars, a list of a number of DEI resources useful for teaching and learning was compiled (supplemental Appendix F).

#### MLA Reads Virtual Book Discussion Group.

The MLA Reads Virtual Book Discussion Group evolved from the special content session, “Transforming Libraries Using Implicit Bias Training,” which had been presented at MLA '18. Session participants expressed the need for safe spaces to learn, discuss, and process the implications of biases on their work as information professionals and in their personal lives. The virtual book discussion group provided a mechanism for thoughtful discussion on challenging issues and topics in a safe, welcoming, and inclusive environment.

Jones and Bartley, the original organizers of the 2018 special content session, planned and facilitated the first virtual book discussion group for approximately fifty librarians on the topic of implicit bias, using Mahzarin R. Banaji and Anthony G. Greenwald's book, *Blindspot: Hidden Biases of Good People,* as a platform for safe and thought-provoking interactions for discussion. Funding for print books and MLA CE credits for participants was sourced from the National Network of Libraries of Medicine (NNLM) Southeastern Atlantic Region (SEA). Six sessions were held, beginning in December 2018 with a welcome session, followed by four ninety-minute virtual sessions, and culminating with an on-site session at MLA '19. Group facilitators were identified to host eight groups, with an additional facilitator for make-up sessions. They were Jones, Bartley, Melissa De Santis, AHIP, Charlene Finley, Ryan Harris, AHIP, Kathryn Houk, AHIP, Don P. Jason III, Annabelle V. Nuñez, Virginia (Ginny) Pannabecker, AHIP, and Dede Rios, AHIP.

The second book discussion took place over four sessions from November 2019 through February 2020. Continuing the conversation on implicit bias, the book chosen for the discussion was *The Person You Mean to Be: How Good People Fight Bias* by Dolly Chugh. De Santis was able to secure funding from the Midcontinental Region (MCR) of NNLM and sponsorship from the MLA Midcontinental Chapter (MCMLA). De Santis led the organization of the discussion group, along with Jones. The virtual discussion group completed activities in February 2020, with 152 participants and 29 facilitators assigned to 18 scheduled groups, and 2 make-up groups were available for those who could not meet with their assigned groups.

A book chapter with the same title is currently being finalized for publishing in the book, *Implementing Excellence in Diversity, Equity, and Inclusion: A Handbook for Academic Libraries*, to be published by the Association of College & Research Libraries. Authors are some of the original book discussion facilitators: Jones, Bartley, De Santis, Harris, Jason, and Rios.

#### Membership survey.

To meet the MLA goal of gathering membership details and feedback about DEI-related demographics and issues in MLA, the DITF conducted a survey. The purpose was to capture a wide range of demographic information and to understand how the membership feels about DEI in the organization. The survey ran for two weeks in October 2019, using SurveyMonkey as the instrument. The survey's results have been distributed as a preprint in *MLAConnect* and as an article in the *Journal of the Medical Library Association* [6, 7].

## CONCLUSION

A newly formed DEI Committee has been appointed as of June 1, 2020. The committee will continue to work on the unmet goals from the MLA Strategic Plan related to DEI over the next year. Accordingly, the DITF's five goals were to:

1. build activities and programs that create and sustain diverse, inclusive, and welcoming cultures and practices;
2. ensure that members, volunteers, and staff have a high level of awareness of issues related to diversity and inclusion;
3. ensure that what we do as an organization, and how we do it, reflects the essential values of diversity and inclusion;
4. attract a diverse community of members that reflects the diversity of the profession and those we serve; and
5. apply the best practices of professional associations with regard to diversity and inclusion.

As demonstrated by the 2019 membership survey results, gaps remain regarding member satisfaction with MLA and the DEI environment [7]. Although the DITF's initiatives had a positive impact, there is much ongoing work to be done, and the new DEI Committee hopes to carry the DITF's work forward.

## SUPPLEMENTAL FILES

**Appendix A:** Medical Library Association (MLA) strategic plan with diversity and inclusion goal

**Appendix B:** Email call to apply to the Diversity and Inclusion Task Force (DITF)

**Appendix C:** Blog post content by 2017/18 President Barbara A. Epstein, AHIP, FMLA

**Appendix D:** MLA Board of Directors motion text

**Appendix E:** Pronouns

**Appendix F:** Diversity, equity, and inclusion resource list
