# Supplementary material for: Medical Library Association Diversity and Inclusion Task Force Report
Source: J Med Libr Assoc. 2021 Jan 1;109(1):141–53. doi: 10.5195/jmla.2021.1112 (PMC7772976; doi:10.5195/jmla.2021.1112)
Supplement: Supplementary file 1 — Appendix A: Medical Library Association (MLA) strategic plan with diversity and inclusion goal [file jmla-109-1-141-s01.pdf]

JMLA Journal of the Medical Library Association

SC

Supplemental content to
J Med Libr Assoc. Jan;109(1):dx.doi.org/10.5195/jmla.2021.1112
www.jmla.mlanet.org
© Morgan-Daniel, Goodman, Franklin, Bartley, Noe, Pionke 2021

Medical Library Association Diversity and Inclusion Task Force Report

Jane Morgan-Daniel, AHIP; Xan Y. Goodman, AHIP; Sandra G. Franklin, AHIP, FMLA; Kelsa Bartley; Matthew Nicholas Noe; JJ Pionke

APPENDIX A

Medical Library Association (MLA) strategic plan with diversity and inclusion goal

Goal: Diversity and Inclusion

Created May 2017, expected completion by May 2019; extended to May 2020

The committee shall be responsible for the execution of the goal defined as follows:

Evaluate and improve MLA practices as they relate to diversity and inclusion in MLA in areas such as:

- MLA defining documents, including vision, mission, values, and code of ethics statements;
- MLA publications, including the *Journal of the Medical Library Association* and *MLAConnect;*
- MLA programs, including the annual meeting, education, and credentialing;
- formulating position statements, including scope of issues and processes;
- engaging members in a constructive discourse on sensitive issues; and
- encouraging a diverse audience to participate in MLA leadership

in order to:

1. build activities and programs that create and sustain diverse, inclusive, and welcoming cultures and practices;
2. ensure that members, volunteers, and staff have a high level of awareness of issues related to diversity and inclusion;
3. ensure that what we do as an organization, and how we do it, reflects the essential values of diversity and inclusion;
4. attract a diverse community of members that reflects the diversity of the profession and those we serve; and
5. apply the best practices of professional associations with regard to diversity and inclusion.

The committee will reach out to members; coordinate efforts with existing MLA entities such as MLA committees, caucuses, domain hubs, and headquarters; and make recommendations to the board.
