# Supplementary material for: Medical Library Association Diversity and Inclusion Task Force Report
Source: J Med Libr Assoc. 2021 Jan 1;109(1):141–53. doi: 10.5195/jmla.2021.1112 (PMC7772976; doi:10.5195/jmla.2021.1112)
Supplement: Supplementary file 2 — Appendix B: Email call to apply to the Diversity and Inclusion Task Force (DITF) [file jmla-109-1-141-s02.pdf]

Journal of the Medical Library Association

Supplemental content to
J Med Libr Assoc. Jan;109(1):dx.doi.org/10.5195/jmla.2021.1112
www.jmla.mlanet.org
© Morgan-Daniel, Goodman, Franklin, Bartley, Noe, Pionke 2021

Medical Library Association Diversity and Inclusion Task Force Report

Jane Morgan-Daniel, AHIP; Xan Y. Goodman, AHIP; Sandra G. Franklin, AHIP, FMLA; Kelsa Bartley; Matthew Nicholas Noe; JJ Pionke

APPENDIX B

Email call to apply to the Diversity and Inclusion Task Force (DITF)

The MLA president (Barbara A. Epstein, AHIP, FMLA) will appoint a new Diversity and Inclusion Task Force to reach out to members; coordinate efforts with existing MLA entities such as MLA committees, sections, special interest groups (SIGs), and headquarters; and make recommendations to the board.

If you would like to be considered for appointment on the new task force, please complete the application below. Consider applying for this task force if:

- You can commit to two or three years of work
- You can spend up an average of ten hours each month contributing to the initiative
- You have a deep understanding of the issues of diversity and inclusion in an organizational setting
- You are passionate about making this MLA strategic goal a success

Please indicate whether you can attend the MLA annual meeting to meet with task force members and connect with membership.

We expect that there will be many more applicants for the task force than can be appointed. If you are not appointed to the task force, please note that there will be multiple ways to contribute to this important initiative, as the task force will be reaching out to all members directly and through existing sections, SIGs, and other MLA groups.

Would like to be considered as a member of the Diversity and Inclusion Task Force?

Yes

What are the 3 most important reasons you want to participate in the task force?

Briefly describe (100 words or less) key experience areas that would make you an outstanding contributor to the task force.

I plan to attend the next two MLA annual meetings

Yes

No

Unsure
