# Supplementary material for: Medical Library Association Diversity and Inclusion Task Force Report
Source: J Med Libr Assoc. 2021 Jan 1;109(1):141–53. doi: 10.5195/jmla.2021.1112 (PMC7772976; doi:10.5195/jmla.2021.1112)
Supplement: Supplementary file 3 — Appendix C: Blog post content by 2017/18 President Barbara A. Epstein, AHIP, FMLA [file jmla-109-1-141-s03.pdf]

JMLA Journal of the Medical Library Association

SC
Supplemental content to
J Med Libr Assoc. Jan;109(1):dx.doi.org/10.5195/jmla.2021.1112
www.jmla.mlanet.org
© Morgan-Daniel, Goodman, Franklin, Bartley, Noe, Pionke 2021

Medical Library Association Diversity and Inclusion Task Force Report

Jane Morgan-Daniel, AHIP; Xan Y. Goodman, AHIP; Sandra G. Franklin, AHIP, FMLA; Kelsa Bartley; Matthew Nicholas Noe; JJ Pionke

APPENDIX C

Blog post content by 2017/18 President Barbara A. Epstein, AHIP, FMLA

Newly Appointed Diversity Task Force Announced

August 2017

https://www.mlanet.org/p/bl/ar/blogaid=1636

At MLA ’17 in Seattle, the MLA Board of Directors approved a new strategic goal to evaluate and improve MLA practices as they relate to diversity and inclusion in our mission, values, ethics, publications, and activities. We want to build programs that are welcoming and inclusive, attract a diverse community of members, and ensure that what we do as an organization and how we do it reflect these essential values.

To achieve this goal, we created a two-year Diversity and Inclusion Task Force to lead this initiative, and we issued a call for interested MLA members to apply. The response from our members was overwhelmingly positive, with nearly forty applications. While not everyone could be appointed as a member, we anticipate that there will be other opportunities to contribute as the task force gets underway.

I am pleased to announce that Sandra G. Franklin, AHIP, FMLA, will chair this task force. Task force members—representing a wide array of backgrounds, experiences and interests—are:

- Kelly Akin
- Diana Almader-Douglas
- Kelsa Bartley
- Xan Goodman
- Stephen Kiyoi, AHIP
- Jane Morgan-Daniel
- Matthew Nicholas Noe
- JJ Pionke
- Neville D. Prendergast
- Gurpreet Kaur Rana
- Jessica Sender, AHIP
- Amy Taylor
- Beverly Murphy, AHIP, FMLA, board liaison
- Kevin Baliozian, MLA executive director

In launching this initiative, I have had the opportunity to review the many activities related to diversity and inclusion occurring throughout the association, including our chapters, special interest groups (SIGs), and sections. For example, our newly adopted professional competencies include a performance indicator for measuring how well each of us “integrates multicultural awareness and

JMLA Journal of the Medical Library Association

Supplemental content to
J Med Libr Assoc. Jan;109(1):dx.doi.org/10.5195/jmla.2021.1112
www.jmla.mlanet.org
© Morgan-Daniel, Goodman, Franklin, Bartley, Noe, Pionke 2021

appreciation of diversity and equality into professional practice." The Mid-Atlantic Chapter (MAC) has its own diversity task force that is surveying MAC members to assess diversity issues in the chapter. MLA '17 in Seattle included a special content session, "Call to Action for Diversity and Inclusion: Perspectives for Our Patrons," and there was a chapter sharing roundtable on "Diversity and Inclusion in Libraries." MLA's Oral History Project has conducted histories of health sciences librarians from diverse ethnic and global communities. The current MLA Board is one of the most diverse groups ever, and Beverly Murphy will be the organization's first African American president when she assumes office next May.

So we have taken the first steps in what will be an ongoing effort. I look forward to the leadership of this new task force creating a roadmap for moving forward, and I appreciate the generosity of our members in volunteering to participate.
