# Supplementary material for: Medical Library Association Diversity and Inclusion Task Force Report
Source: J Med Libr Assoc. 2021 Jan 1;109(1):141–53. doi: 10.5195/jmla.2021.1112 (PMC7772976; doi:10.5195/jmla.2021.1112)
Supplement: Supplementary file 4 — Appendix D: MLA Board of Directors motion text [file jmla-109-1-141-s04.pdf]

JMLA Journal of the Medical Library Association

Supplemental content to
J Med Libr Assoc. Jan;109(1):dx.doi.org/10.5195/jmla.2021.1112
www.jmla.mlanet.org
© Morgan-Daniel, Goodman, Franklin, Bartley, Noe, Pionke 2021

Medical Library Association Diversity and Inclusion Task Force Report

Jane Morgan-Daniel, AHIP; Xan Y. Goodman, AHIP; Sandra G. Franklin, AHIP, FMLA; Kelsa Bartley; Matthew Nicholas Noe; JJ Pionke

APPENDIX D

MLA Board of Directors motion text

The Diversity and Inclusion Task Force (DITF) crafted text to submit to the MLA Board in May 2019. Motion text covered MLA defining documents.

Diversity and Inclusion Task Force motion

Motion on language

Director Gupreet Kaur Rana presented the DITF motion on language to the board. The board discussed the motion and had several questions and suggestions concerning the specific language on some of the MLA statements.

It was:

MOVED, that MLA adopt the following language in MLA documents, such as websites, awards, scholarships and fellowship descriptions, and MLA communications:

- Latinx, hereby replacing terms such as Hispanic, Latino, or Latina
- Underrepresented, hereby replacing terms such as Minority
- They/them/theirs, hereby replacing terms such as she/her/hers and he/him/his
- Mx as an additional profile prefix

and include diversity, equity, inclusion, and gendered language as needed throughout those documents;

FURTHER MOVED, that the board amend the MLA Vision statement by adding the terms diversity, equity and inclusion:

CURRENT LANGUAGE (This was the current language at the time the motion was submitted).

Our dreams and aspirations. What we strive to be. Our desired future state.

The Medical Library Association (MLA) believes that quality information is essential for improved health. MLA aspires to be the association of the most visible, valued, and trusted health information experts. To that end, MLA fosters excellence in the professional practice and leadership of health sciences library and information professionals in order to enhance the quality of health care, education, and research throughout the world.

PROPOSED LANGUAGE:

Our dreams and aspirations. What we strive to be. Our desired future state.

The Medical Library Association (MLA) believes that quality information is essential for improved health. MLA aspires to be the association of the most visible, valued, and trusted health information experts. MLA strives to enhance the quality of health care, education, and research throughout the world. To that end, MLA fosters excellence and is committed to diversity, equity, and inclusion in professional practice, leadership of health sciences libraries, and information professionals, now and in the future.

JMLA Journal of the Medical Library Association

SC

Supplemental content to
J Med Libr Assoc. Jan;109(1):dx.doi.org/10.5195/jmla.2021.1112
www.jmla.mlanet.org
© Morgan-Daniel, Goodman, Franklin, Bartley, Noe, Pionke 2021

FURTHER MOVED, that the board amend the MLA Mission statement by adding "Promoting diverse and inclusive library personnel who are reflective of the society we serve" as a bullet 4 to part 1.

<u>CURRENT LANGUAGE:</u> (This was the current language at the time the motion was submitted)

Recruitment, Membership, and Leadership in the Profession

MLA to

- Recruit to and retain individuals in the profession
- Serve members
- Develop leadership skills

By

- Promoting health sciences librarianship
- Promoting exploration of new roles, knowledge, and skills
- Promoting mentorship and leadership
- Promoting diverse and inclusive library personnel who are reflective of the society we serve

<u>PROPOSED LANGUAGE</u> [as recommended by MLA Board, May 2019]:

By

- Promoting health sciences librarianship
- Promoting exploration of new roles, knowledge, and skills
- Promoting mentorship and leadership
- Promoting inclusive policies resulting in a diverse library workforce reflective of the communities we serve

FURTHER MOVED, that the board amend the MLA Core Values statement by adding bullets 5 and 6 as follows:

<u>CURRENT LANGUAGE</u> (This was the current language at the time the motion was submitted)

- Use of scientific evidence in making health care decisions
- Public awareness of, access to, and use of high-quality health information
- Lifelong learning and professional development
- Advancement of health information research and evidence-based practice
- Community and collaboration within and outside the profession
- Irreproachable ethical standards

<u>PROPOSED LANGUAGE ADDITION (BULLETS 5 AND 6 ONLY):</u>

- Use of scientific evidence in making health care decisions
- Public awareness of, equitable access to, and use of high-quality health information
- Lifelong learning and professional development
- Advancement of health information research and evidence-based practice
- An open, inclusive, and collaborative environment within and outside the profession
- Advancement and support of accessibility for all stakeholder groups
- Irreproachable ethical standards

FURTHER MOVED, that the board amend the MLA Code of Ethics by adding "regardless of race, ethnicity, gender and gender identity, age, sexual orientation, socioeconomic status, language, culture, nationality, age, (dis)ability status, or religion" to the profession section:

JMLA Journal of the Medical Library Association

Supplemental content to
J Med Libr Assoc. Jan;109(1):dx.doi.org/10.5195/jmla.2021.1112
www.jmla.mlanet.org
© Morgan-Daniel, Goodman, Franklin, Bartley, Noe, Pionke 2021

CURRENT LANGUAGE (This was the current language at the time the motion was submitted)

Profession

- The health sciences librarian advances and upholds the philosophy and ideals of the profession.
- The health sciences librarian advocates and advances the knowledge and standards of the profession.
- The health sciences librarian conducts all professional relationships with courtesy and respect.
- The health sciences librarian maintains high standards of professional integrity.

PROPOSED LANGUAGE:

- The health sciences librarian conducts all professional relationships with courtesy and respect, regardless of race, ethnicity, gender and gender identity, age, sexual orientation, socioeconomic status, political beliefs, language, culture, nationality, age, ability status, or religion.

[END OF MOTION]

SEE DEI DEFINITION AS REQUESTED.

Defining diversity, equity, and inclusion in the MLA Context:

> Diversity, equity, and inclusion
> are the threads that strengthen the fabric of
> the Medical Library Association.

Diversity, equity, and inclusion (DEI) are essential values of the Medical Library Association (MLA). To that end, MLA fosters excellence and is committed to diversity, equity, and inclusion in professional practice, leadership of health sciences libraries, and information professionals, now and in the future.

How does MLA interpret *diversity*, *equity* and *inclusion*?

Diversity: ways that people are both alike and different; understanding, accepting, and valuing differences that include race and ethnicity, gender and gender identity, sexual orientation, socioeconomic status, political beliefs, language, culture, nationality, age, (dis)ability status, and religion

Equity: takes differences into account to ensure fair and impartial processes and outcomes, and equal opportunity

Inclusion: all individuals are treated fairly and respectfully; are intentionally valued for their distinctive skills, experiences, and perspectives; have equal access to resources and opportunities; feel a sense of belonging; and can contribute fully to the association's success.

Adapted from ALA ODLOS Glossary of Terms [1], University of Michigan: Diversity, Equity and Inclusion: Defining DEI [2], Racial Equity Tools: Glossary [3], Built in [4].

REFERENCES

1. American Library Association, Office for Diversity, Literacy and Outreach Services (ODLOS). ODLOS glossary of terms [Internet]. The Association [cited 21 Aug 2019]. <http://www.ala.org/aboutala/odlos-glossary-terms>.
2. University of Michigan, Defining DEI: diversity, equity and inclusion [Internet]. The University [cited 21 Aug 2019]. <https://diversity.umich.edu/about/defining-dei/>.

JMLA Journal of the Medical Library Association

Supplemental content to
J Med Libr Assoc. Jan;109(1):dx.doi.org/10.5195/jmla.2021.1112
www.jmla.mlanet.org
© Morgan-Daniel, Goodman, Franklin, Bartley, Noe, Pionke 2021

3. Racial Equity Tools: Glossary: about the racial equity tools glossary [Internet]. Racial Equity Tools[cited 21 Aug 2019]. <https://www.racialequitytools.org/glossary>.
4. Built in. Diversity + inclusion [Internet]. Built in [cited 21 Aug 2019]. <https://builtin.com/diversity-inclusion>.

One minor change in motion language

Both motions passed, there was a minor change to one motion:

Only one small change was made to the language to use “ability status” in place “(dis)ability status” as such: “regardless of race, ethnicity, gender and gender identity, age, sexual orientation, socioeconomic status, political beliefs, language, culture, nationality, age, ~~(dis)ability~~ ability status, or religion.”
