# Supplementary material for: Medical Library Association Diversity and Inclusion Task Force Report
Source: J Med Libr Assoc. 2021 Jan 1;109(1):141–53. doi: 10.5195/jmla.2021.1112 (PMC7772976; doi:10.5195/jmla.2021.1112)
Supplement: Supplementary file 5 — Appendix E: Pronouns [file jmla-109-1-141-s05.pdf]

JMLA Journal of the Medical Library Association

Supplemental content to
J Med Libr Assoc. Jan;109(1):dx.doi.org/10.5195/jmla.2021.1112
www.jmla.mlanet.org
© Morgan-Daniel, Goodman, Franklin, Bartley, Noe, Pionke 2021

Medical Library Association Diversity and Inclusion Task Force Report

Jane Morgan-Daniel, AHIP; Xan Y. Goodman, AHIP; Sandra G. Franklin, AHIP, FMLA; Kelsa Bartley; Matthew Nicholas Noe; JJ Pionke

APPENDIX E

Pronouns

For example, the registration form may ask the following:

What pronouns do you prefer colleagues use when talking about you? Pronouns will appear on your badge.

☐ She/her/hers

☐ He/him/his

☐ They/them/theirs

☐ Other: Please specify: ________________________ (15 character limit)

Diversity and inclusivity are important to MLA. At MLA '19, attendees will have the option of selecting personal pronouns on the registration form, and your pronouns will be visible on meeting badges.

People identify on a broad spectrum of gender identities, including male, female, nonbinary, and more. It is important to remember that gender identity is *not* visible. It is an *internal* sense of one's own gender. While many people feel that their gender identity aligns with the sex assigned to them at birth, this is not true for many others.

Using a person's chosen name and desired pronouns is a form of mutual respect and courtesy. You cannot always know someone's gender identity just by looking at them, which is why sharing and making pronouns visible is important.

A culture that readily asks about or provides pronouns is one committed to reducing the risk of disrespect and embarrassment for all community members. MLA is building an inclusive culture for all of our members, and adding the option of sharing personal pronouns on meeting badges is a good step in that direction.
