# Supplementary material for: Medical Library Association Diversity and Inclusion Task Force Report
Source: J Med Libr Assoc. 2021 Jan 1;109(1):141–53. doi: 10.5195/jmla.2021.1112 (PMC7772976; doi:10.5195/jmla.2021.1112)
Supplement: Supplementary file 6 — Appendix F: Diversity, equity, and inclusion resource list [file jmla-109-1-141-s06.pdf]

JMLA Journal of the Medical Library Association

SC
Supplemental content to
J Med Libr Assoc. Jan;109(1):dx.doi.org/10.5195/jmla.2021.1112
www.jmla.mlanet.org
© Morgan-Daniel, Goodman, Franklin, Bartley, Noe, Pionke 2021

Medical Library Association Diversity and Inclusion Task Force Report

Jane Morgan-Daniel, AHIP; Xan Y. Goodman, AHIP; Sandra G. Franklin, AHIP, FMLA; Kelsa Bartley; Matthew Nicholas Noe; JJ Pionke

APPENDIX F

Diversity, equity, and inclusion resource list

Below is an unannotated list of recommended readings, conversation outlets, and online training related to diversity, equity, and inclusion.

Read

Bongiovanni A, Jimerson T. A quick & easy guide to they/them pronouns. Portland, OR: Limerence Press; 2018.

Chou R, Pho A, eds. Pushing the margins: women of color and intersectionality in LIS. Sacramento, CA: Litwin Books & Library Juice Press; 2018.

Czerwiec MK, Williams I, Squier SM, Green MJ, Myers KR, Smith ST. Graphic medicine manifesto. University Park, PA: Penn State University Press; 2020.

Forney E. Marbles: mania, depression, Michelangelo, and me. London, UK: Penguin Random House; 2013.

Jack AA. The privileged poor: how elite colleges are failing disadvantaged students. Cambridge, MA: Harvard University Press; 2019.

Jones SD, Murphy B, eds. Diversity and inclusion in libraries: a call to action and strategies for success. Lanham, MD: Rowman & Littlefield; 2019. Medical Library Association Books Series.

Kendi IX. How to be antiracist. New York, NY: One World; 2019.

Kobabe M. Gender queer: a memoir. St. Louis, MO: Lion Forge; 2014.

Piepmeier O, Grimm S, eds. Comics and critical librarianship: reframing the narrative in academic libraries. Sacramento, CA: Litwin Books & Library Juice Press; 2019.

Taylor W, Kindred C. African-Americans are more likely to distrust the medical system. blame the Tuskegee Experiment. The Nib [Internet]. 26 Feb 2018 [cited 25 Sep 2020]. <https://thenib.com/tuskegee-experiment?t=default>.

Zuckerberg MG, Zuckerberg JR. A quick & easy guide to queer & trans identities. Portland, OR: Limerence Press; 2019.

Conversation

Follow Matthew Nicholas Noe on Twitter at https://twitter.com/NoetheMatt

Follow conversations about critical librarianship on Twitter using the hash tag #critlib

JMLA Journal of the Medical Library Association

SC

Supplemental content to
J Med Libr Assoc. Jan;109(1):dx.doi.org/10.5195/jmla.2021.1112
www.jmla.mlanet.org
© Morgan-Daniel, Goodman, Franklin, Bartley, Noe, Pionke 2021

Online training

Medical Library Association. Diversity, equity, and inclusion: making MLA a more diverse and inclusive association [Internet]. The Association [cited 25 Sep 2020]. <https://www.mlanet.org/DiversityandInclusion>.

National Network of Libraries of Medicine. Diversity, equity, and inclusion: nine conversations that matter to health sciences librarians with Jessica Pettitt [Internet]. The Network [cited 25 Sep 2020]. <https://nnlm.gov/classes/DEI>.

National Network of Libraries of Medicine. NNLM clinical conversations training program: cultural humility [Internet]. The Network [cited 25 Sep 2020]. <https://nnlm.gov/mar/guides/clinical-conversations-training-program/cultural-humility>.

Pashia A, Ivory CJ. Examining institutional racism in libraries [Internet]. Library Juice Academy [cited 25 Sep 2020]. <https://libraryjuiceacademy.com/shop/course/193-examining-institutional-racism-libraries/>.

Project Implicit. Preliminary information [Internet]. The Project [cited 25 Sep 2020]. <https://implicit.harvard.edu/implicit/takeatest.html>.
